# Prognostic Value of EGFR Mutation and ERCC1 in Patients with Non-Small Cell Lung Cancer Undergoing Platinum-Based Chemotherapy

**DOI:** 10.1371/journal.pone.0071356

**Published:** 2013-08-05

**Authors:** Fumie Yamashita, Koichi Azuma, Tsukasa Yoshida, Kazuhiko Yamada, Akihiko Kawahara, Satoshi Hattori, Hiroaki Takeoka, Yoshiaki Zaizen, Tomotaka Kawayama, Masayoshi Kage, Tomoaki Hoshino

**Affiliations:** 1 Division of Respirology, Neurology, and Rheumatology, Department of Internal Medicine, Kurume University School of Medicine, Kurume, Fukuoka, Japan; 2 Department of Diagnostic Pathology, Kurume University Hospital, Kurume, Fukuoka, Japan; 3 Biostatistics Center, Kurume University, Kurume, Fukuoka, Japan; University of Central Florida, United States of America

## Abstract

**Background:**

In order to improve the outcome of patients with non-small cell lung cancer (NSCLC), a biomarker that can predict the efficacy of chemotherapy is needed. The aim of this study was to assess the role of EGFR mutations and ERCC1 in predicting the efficacy of platinum-based chemotherapy and the outcome of patients with NSCLC.

**Methods:**

We conducted a retrospective study to analyze the relationships between EGFR mutations or ERCC1 expression and progression-free survival (PFS) in patients with NSCLC who received platinum-based chemotherapy. EGFR mutation status was determined using the peptide nucleic acid-locked nucleic acid polymerase chain reaction clamp method, and immunohistochemistry was used to examine the expression of ERCC1 in tumor samples obtained from the patients.

**Results:**

Among the NSCLC patients who received platinum-based chemotherapy, the median PFS was significantly better in those who had never smoked and those with exon 19 deletion, and the median overall survival (OS) was significantly better in those who had never smoked, those with exon 19 deletion, and women. Cox regression analysis revealed that exon 19 deletion and having never smoked were significantly associated with both PFS and OS. Subset analysis revealed a significant correlation between ERCC1 expression and EGFR mutation, and ERCC1-negative patients with exon 19 deletion had a longer PFS than the other patients; ERCC1-positive patients without exon 19 deletion had a shorter PFS than the other patients.

**Conclusions:**

Our results indicate that among NSCLC patients receiving platinum-based chemotherapy, those with exon 19 deletion have a longer PFS and OS. Our findings suggest that platinum-based chemotherapy is more effective against ERCC1-negative and exon 19-positive NSCLC.

## Introduction

Lung cancer is the leading cause of cancer death worldwide [1]. The epidermal growth factor receptor (EGFR) is considered to be an important molecular target in lung cancer therapy. Somatic activating mutations of the *EGFR* gene have been identified as a major determinant of the clinical response to EGFR tyrosine kinase inhibitors (TKIs) such as gefitinib and erlotinib in patients with NSCLC. Most of these mutations occur in exons 19 to 21, which encode the tyrosine kinase domain of the receptor, the most common being deletions in exon 19 (such as delE746-A750) and the L858R point mutation in exon 21. These mutations are found more frequently in female patients, in individuals who have never smoked, and in patients of East Asian ethnicity [2]–[5]. Prospective clinical trials of EGFR-TKI treatment for NSCLC patients with activating EGFR mutations, such as delE746-A750 (exon 19) and L858R (exon 21), have demonstrated high clinical response rates of approximately 80% [4]–[7]. In the IPASS study comparing gefitinib with carboplatin and paclitaxel as the first-line therapy in Asian patients, NSCLC patients with EGFR mutation had a higher response rate than patients without EGFR mutations when they received carboplatin and paclitaxel. The efficacy of platinum-based chemotherapy was better in patients who were positive for EGFR mutation than in those who were negative [8]. These results suggest that NSCLC patients with EGFR mutations may tend to respond to cytotoxic regimens such as platinum-based chemotherapy. Some researchers have reported that patients with EGFR mutations respond to conventional chemotherapy [9]–[13]. However, the mechanisms underlying these responses to chemotherapy have remained unclear.

On the other hand, ERCC1 is a component of the nucleotide excision repair pathway, which is essential for the repair of platinum-DNA adducts and is associated with resistance to platinum-based chemotherapy [13]. Some clinical studies have demonstrated that overexpression of ERCC1 tends to be associated with resistance to platinum-based chemotherapy [14]–[15]. Previously, we also reported that ERCC1 expression estimated by immunohistochemistry was an independent prognostic factor in terms of both proression-free survival (PFS) and overall survival (OS) in patients with NSCLC relapse who had received platinum-based chemotherapy [16]. Overall, NSCLC patients with a low level of ERCC1 had longer survival after platinum-based chemotherapy than those with a high level of ERCC1. Gandara et al. have reported a significant association between EGFR mutation and a low level of ERCC1 mRNA in NSCLC tumor samples [17]. These important findings prompted us to investigate the correlations between EGFR mutations and ERCC1 in NSCLC patients receiving platinum-based chemotherapy. We conducted a retrospective study to assess the utility of EGFR mutation as a predictor of the efficacy of platinum-based chemotherapy and the outcome of patients with NSCLC. Moreover, we used immunohistochemistry to examine the expression of ERCC1 in tumor samples from NSCLC patients receiving platinum-based chemotherapy, and analyzed the relationships between ERCC1 and EGFR mutations in tumors and survival time to determine whether or not the expression of these molecules could be used to predict PFS and OS.

## Materials and Methods

### NSCLC Patients and Treatment

This retrospective study included cases of histologically or cytologically diagnosed NSCLC, which were advanced (stage IIIB or IV) or recurrent at initial diagnosis. We screened 206 NSCLC patients treated with gefitinib or erlotinib between September 2002 and March 2011 at Kurume University Hospital. Among these patients, 103 received platinum-based chemotherapy and EGFR-TKIs. Treatment was continued until the appearance of unacceptable adverse events, disease progression or death, or withdrawal from the study. Clinical information about each case was obtained from the medical records; the parameters included gender, age, Eastern Cooperative Oncology Group (ECOG) performance status (PS), tumor histology, stage, smoking status, and the number of prior chemotherapy courses. Patient characteristics noted included sex, age, PS, tumor histology, disease stage, postoperative disease recurrence, smoking history, prior chemotherapy, and type of EGFR mutation.

Tumor response was examined by computed tomography and evaluated according to the Response Evaluation Criteria in Solid Tumors (RECIST). Response was confirmed at least 4 weeks (for a complete or partial response) or 6 weeks (for stable disease) after it was first documented. This study was approved by the institutional review board of Kurume University.

### DNA Extraction and PNA-LNA PCR Clamp Assay

For *EGFR* mutation analysis, the peptic nucleic acid-locked nucleic acid (PNA-LNA) polymerase chain reaction (PCR) clamp method was adopted, using protocols described previously [18]. Specific PNA-LNA probe sets for two mutation sites, exon 19 (delE746-A750) and exon 21 (L858R), were developed and these covered >90% of *EGFR* mutations reported previously in Japan. In brief, genomic DNA was purified from paraffin-embedded tissues using a QIAamp DNA Micro kit (QIAGEN). The PCR primers employed were synthesized by Invitrogen Inc. PNA clamp primers and LNA mutant probes were purchased from FASMEC (Kanagawa, Japan) and IDT (Coralville, IA), respectively. PNA-LNA PCR clamp assay was performed using the SDS-7500 System (Applied Biosystems). Exon sequences for the EGFR kinase domain were amplified by nested PCR using a specific primer.

### Immunohistochemical Evaluation of Paraffin-embedded Tumor Tissues

Adequate tumor specimens were available for 51 of these patients and subjected to ERCC1 detection, as reported previously [16]. Tissue samples were fixed in 10% neutral-buffered formalin and embedded in paraffin. Paraffin sections were then cut at a thickness of 4 µm, mounted on coated glass slides, and labeled with an antibody against ERCC1 (clone 8F1; Neomarkers, Fremont, CA). After the sections had been deparaffinized in xylene and dehydrated in a graded ethanol series, endogenous peroxidase activity was blocked with H_2_O_2_ in methanol. For ERCC1 staining, antigens were retrieved by heating in citric acid (pH 6.0) for 10 min. Sections were incubated with blocking solution (10% Block Ace; Yukijirushi, Tokyo, Japan) and then reacted with anti-ERCC1 monoclonal antibody. After excess antibody had been washed out with PBS, the samples were incubated with horseradish peroxidase (HRP)-labeled goat anti-mouse or anti-rabbit antibody (Nichirei, Tokyo, Japan) for 60 min. The reaction was visualized using 3,3′ diaminobenzidine (DAB) as a substrate (DAKO, Glostrup, Denmark), and the slides were counterstained with hematoxylin. Each slide was heat-treated using Target Retrieval Solution, pH 9.0 (DAKO, Glostrup, Denmark) for 30 min, and incubated with the antibody at 4°C overnight. ERCC1 nuclear expression was classified into four categories: score 0, no staining at all <10% of tumor cells; score 1+, faint/barely perceptible partial nuclear expression in >10% of tumor cells; score 2+, weak to moderate staining of the entire nucleus in >10% of tumor cells; and score 3+, strong staining of the entire nucleus in >10% of tumor cells. The extent of immunohistological staining for ERCC1 was defined as follows: scores of 1+, 2+, and 3+ were regarded as positive, and a score of 0 was regarded as negative. All IHC studies were evaluated by two IHC-experienced observers who were unaware of the conditions of the patients (A.K. and M.K.).

### Statistical Analysis

We used Fisher’s exact test to evaluate the significance of relationships between EGFR mutation status and other patient characteristics. For EGFR-TKIs treatment, PFS was calculated from the date of initiation of EGFR-TKIs until the date of disease progression. For platinum-based chemotherapy treatments, PFS was calculated from the date of initiation of platinum-based chemotherapy until the date of disease progression. OS was defined as the period from the date of initiation of platinum-based chemotherapy until the date of death. Patients without disease progression and who were still alive were treated as censored at their last contact for analysis of PFS and OS, respectively. Survival curves for PFS and OS were estimated by the Kaplan-Meier method, and the difference between the curves for patients with and without EGFR mutations was evaluated by the log-rank test. The Cox proportional hazards model was applied to examine whether EGFR mutation was associated with PFS or OS even after adjustment for other prognostic factors. All tests were two-sided, and differences at *P*<0.05 were considered statistically significant. Statistical analysis was performed with JMP version 10.0.0 and SAS version 9.2 software (SAS Institute, Cary, NC).

## Results

### Relationships between EGFR Mutations and Patient Characteristics

The clinical characteristics of the 103 patients are shown in Table 1. All of the patients received platinum-based chemotherapy and had a good PS (0–1). EGFR mutations were identified in 46 patients. With regard to the type of *EGFR* mutation, 26 patients had deletions in exon 19, and 20 patients had the L858R missense mutation in exon 21. Fifty-six patients were females and 51 had never smoked; the median age of the patients overall was 63 (range, 33 to 78) years. Eighty-nine patients had adenocarcinoma. Platinum compounds were used for first-line chemotherapy in most (99/103) of the patients. On the other hands, 4 patients received gefitinib as a first line chemotherapy followed by platinum compounds as second line chemotherapy. The chemotherapy regimens consisted of carboplatin-paclitaxel in 55 patients, carboplatin-gemcitabine in 12, carboplatin-pemetrexed in 4, carboplatin-vinorelbine in 1, cisplatin-vinorelbine in 18, cisplatin-docetaxel in 4, cisplatin-gemcitabine in 4, cisplatin-vinorelbine-gemcitabine in 3, cisplatin-pemetrexed in 1, and cisplatin-etoposide in 1. The median number of chemotherapy cycles was three (range 1–4). In the EGFR mutation-positive group, 17 patients (36.9%) maintained a partial response (PR), 25 (54.3%) maintained stable disease (SD), and 4 (8.8%) maintained progressive disease (PD), whereas in the EGFR mutation-negative group, 10 patients (17.5%) maintained a PR, 28 (49.1%) maintained SD, and 19 (32.2%) maintained PD. Female gender (P = 0.072), tumor response (P = 0.005), and gefitinib treatment (P>0.001) were significantly more frequent in the group of patients with EGFR mutations, whereas other characteristics showed no inter-group differences (Table 1).

**Table 1:** Relation between expression score for EGFR mutants and various patient characteristics.

				EGFR mutation		
Characteristic		Number of patients		L858R or Exon19 deletion	Negative	*P* value^a^
Age (years)						
High (>63)		55		24	31	0.850
Low (<63)		48		22	26	
Sex						
Male		47		16	31	0.072
Female		56		30	26	
Histology						
Adenocarcinoma		89		42	47	0.253
Non-adeno		14		4	10	
Smoking status						
Never-smoker		51		27	24	0.115
Smoker		52		19	33	
Stage						
III		2		1	1	1.000
IV or reccurent		101		45	56	
Response (platinum-compounds)						
PR		27		17	10	0.005
SD		53		25	28	
PD		23		4	19	
Performance status						
0		84		39	45	0.610
1		19		7	12	
Line (EGFR-TKIs line)						
1st line		4		4	0	0.085
2nd line		70		30	40	
3rd line		28		11	17	
4th line		1		1	0	
Treatment						
Gefitinib		75		42	33	0.001>
Erlotinib		28		4	24	

a Determined by Fisher’s exact test.

### Survival Analysis

**Figure 1:**
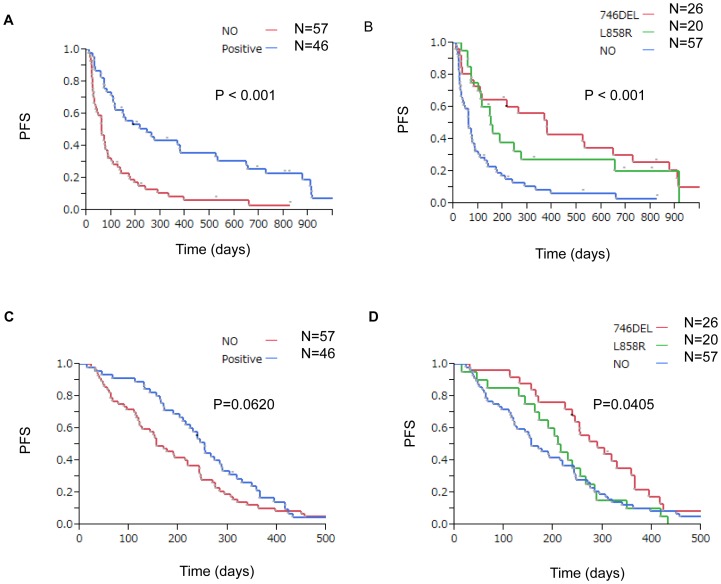
Kaplan-Meier survival analysis of patients with NSCLC with or without EGFR mutations. Differences in progression-free survival and overall survival between subgroups were analyzed by the log-rank test. A, B: For EGFR-TKIs treatment, the median PFS was significantly longer in EGFR-mutation positive than in EGFR mutation-negative patients (P<0.001, P<0.001) C: Among patients with NSCLC who received platinum-based chemotherapy, the median PFS was significantly better for those with EGFR mutations than for those without. D: Among patients with NSCLC who received platinum-based chemotherapy, the median PFS was significantly better for those with exon 19 deletion or L858R.

The median duration of follow-up was 565 (range 31–3367) days. Among 103 NSCLC patients who received platinum-based chemotherapy, 101 suffered disease progression and 73 died. For EGFR-TKIs treatment, the median PFS was significantly longer in EGFR mutation-positive than in EGFR mutation-negative cases (P<0.001, P<0.001) (Fig. 1A and 1B), whereas there was no significant difference in median OS between the two groups (*P* = 0.126, P = 0.122), respectively.

Among patients with NSCLC who received platinum-based chemotherapy, the median PFS was significantly better for those who had never smoked (P = 0.003) and for those with exon 19 deletion or L858R (P = 0.040) (Fig. 1C and Fig. 1D). However, PFS was not associated with other factors, such as age, sex, histology, stage, and EGFR-TKIs treatment (Table 2). Also among patients with NSCLC who received platinum-based chemotherapy, the median OS was significantly better for female patients (P = 0.020), patients who had never smoked (P = 0.03), patients with stage IV or recurrent disease (P = 0.010), and patients with exon 19 deletion (P = 0.028). However, OS was not associated with other factors, such as age, histology, and EGFR-TKIs treatment (Table2). Finally, Cox regression analysis, including factors shown to be significantly associated with PFS or OS by the log-rank test, revealed that EGFR mutation and smoking status (never having smoked) were significantly associated with PFS (P = 0.036 for heterogeneity among exon 19 deletion, L858R and wild type; P = 0.024 for smoking status with hazard ratio (HR) 0.427 and 95% confidence interval (CI) 0.213–0.892). By pairwise comparison relative to the wild type, patients with exon 19 deletion had significantly longer PFS than those with the wild type (HR, 0.566; 95% CI, 0.340 to 0.911; *P* = 0,019), whereas patients with L858R did not have significantly longer PFS (HR, 1.073; 95% CI, 0.621–1.780) (Table 3). For OS, similar results were obtained: EGFR mutation was significantly associated with OS (P = 0.010 for heterogeneity) and smoking status was close to statistical significance (P = 0.082; HR, 0.523; 95% CI, 0.256–1.086). Patients with exon 19 deletion had significantly longer OS that those with the wild type (HR, 0.452; 95% CI, 0.242 to 0.811; *P* = 0,007), whereas those with L858R did not (HR, 1.176; 95% CI, 0.628–2.100, P = 0.600) (Table 4).

**Table 2:** Factors associated with PFS and OS in patients with NSCLC treated with Platinum doublets.

Factor	Number ofpatients (N)	Median PFS(days)	*P* value^a^	Median OS(days)	*P* value^a^
Age (years)					
High (>63)	55	197.0	0.276	574.0	0.332
Low (<63)	48	241.0		1093.5	
Sex					
Male	47	173.5	0.044	562.5	0.02
Female	56	234.0		1231.5	
Histology					
Adenocarcinoma	89	216.5	0.921	845.0	0.007
Non-adeno	14	184.0		522.5	
Smoking status					
Never-smoker	51	240.5	0.003	1280.5	0.0035
Smoker	52	167.0		562.5	
Stage					
III	2	190.0	0.797	294.0	0.010
Recurrent or IV	101	216.5		718.0	
EGFR mutation					
Exon19 deletion	26	281.0	0.040	1502.0	0.028
L858R	20	211.5		564.5	
Negative	57	153.5		582.5	
Line (EGFR-TKIs line)					
1st	4	121.0	0.985	552.0	0.465
2nd	70	214.0		718.0	
3rd or 4th	29	202.5		660.0	

a Univariate analysis by log-rank test.

**Table 3:** Multivariate analysis of PFS.

Factor	Comparison	HR (95%CI)	P-value
Smoking status	Never smoker/Smoker	0.427 (0.213–0.892)	0.024
Sex	Male/Female	0.742(0.373–1.540)	0.417
Mutation	Exon19 deletion/Wild type	0.566(0.340–0.911)	0.019
	L858R/Wild type	1.073(0.621–1.780)	0.793

**Table 4:** Multivariate analysis of OS.

Factor	Comparison	HR (95% CI)	P-value
Smoking status	Never smoker/Smoker	0.523 (0.256–1.086)	0.082
Sex	Male/Female	1.189 (0.591–2.445)	0.632
Mutation	Exon19 deletion/Wild type	0.452 (0.242–0.811)	0.007
	L858R/Wild type	1.176 (0.628–2.100)	0.600

### Immunohistochemical Assessment of ERCC1 and Correlation between ERCC1 and Survival

**Figure 2:**
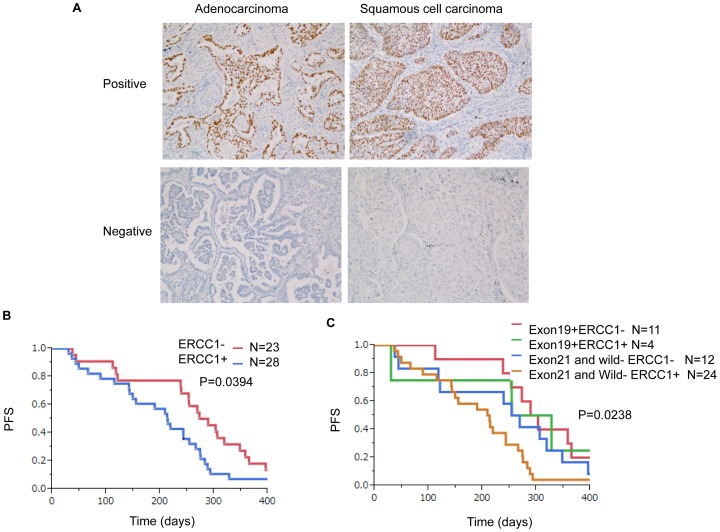
Immunohistochemical assessment of ERCC1 and correlation between ERCC1 and survival. A: Expression of ERCC1 was assessed by immunohistochemistry. Expression of ERCC1 protein was detected in the nuclei of cancer cells (x400). B: Kaplan-Meier survival curves according to ERCC1 expression score. C: ERCC1-negative patients with exon 19 deletion had longer PFS than the others, and ERCC1-positive patients without exon 19 deletion had shorter PFS than the others.

Adequate tumor specimens were available from 51 of these patients, and were subjected to ERCC1 detection. Expression of ERCC1 was assessed by immunohistochemistry (Fig. 2A). Expression of the ERCC1 protein was detected in the nuclei of cancer cells. Table 5 shows the relationships between ERCC1 expression and other clinicopathologic factors. Nuclear expression of ERCC1 was classified into four categories. Twenty-three patients had score 0, 12 had score 1+, 8 had score 2+, and 8 had score 3+ (median 1). Because the median ERCC1 expression score is score 1+, scores of 1+, 2+ and 3+ were regarded as positive and score of 0 as negative. ERCC1 expression was positive in 28 patients and negative in 23. ERCC1 expression was significantly correlated with EGFR mutation (P = 0.030) (Fig. 2B), but not with other factors, including age (*P* = 1.000), sex (*P* = 1.000), histology (*P* = 1.000), smoking status (*P* = 0.773), or response to chemotherapy (*P* = 0.374).

**Table 5:** Relation between expression score for ERCC1 and various patient characteristics.

			ERCC1(scoring 0–3 Median 1)		
Characteristic			Negative (0>)	Positive (1<)	*P* value^a^
Age (years)					
High (>63)		26	12	14	1.000
Low (<63)		25	11	14	
Sex					
Male		19	9	10	1.000
Female		32	14	18	
Histology					
Adenocarcinoma		45	20	25	1.000
Non-adeno		6	3	3	
Smoking status					
Never-smoker		31	13	18	0.773
Smoker		20	10	10	
Response					
PR		13	8	5	0.374
SD		27	11	16	
PD		11	4	7	
EGFR mutation					
Exon19 deletion		15	11	4	0.030
L858R		12	4	8	
Wild type		24	8	16	

a Determined by Fisher’s exact test.

The median PFS was significantly better in patients who had never smoked (P = 0.027) and in patients who were ERCC1-negative (P = 0.039), whereas it was unrelated to other factors, such as age, sex, histology, and EGFR mutation (Table 6). The median OS was significantly better in patients with adenocarcinoma (P = 0.014) and patients who had never smoked (P = 0.040), whereas it was unrelated to other factors such as age, EGFR mutation, and ERCC1 (Table 6). Figure 2C shows Kaplan-Meier plots constructed using a combination of ERCC1 expression and EGFR status. Patients with exon 19 deletion and low ERCC1 expression showed longer PFS, whereas those without exon 19 deletion and showing high expression of ERCC1 had shorter PFS. We examined the effects of mutation status and ERCC1 simultaneously using Cox regression adjusting for factors that had been shown to be significantly associated with PFS or OS by the log-rank test. Cox regression analysis revealed that EGFR mutation and smoking status (having never smoked) were significantly associated with PFS (P = 0.045 for heterogeneity among exon 19 deletion, L858R and wild type; P = 0.013 for smoking). Pairwise comparison relative to the wild type showed that patients with exon 19 deletion had significantly longer PFS than those with the wild type (HR, 0.484; 95% CI, 0.227 to 0.984; *P* = 0.045), whereas patients with L858R did not have significantly longer PFS (HR, 1.050; 95%CI, 0.486–2.163; P = 0.897) (Table 7). As suggested by Figure 2C, Cox regression revealed that ERCC1-negative patients with exon 19 deletion had longer PFS, whereas ERCC1-positive patients without exon 19 deletion had shorter PFS than the others. On the other hand, neither ERCC1 expression nor mutation status was significantly associated with OS. Cox regression analysis, including factors shown to be significantly associated with OS by the log-rank test, revealed that having never smoked and having adenocarcinoma were significantly associated with OS (P = 0.021 for smoking status, P = 0.047 for histology). However, no significant associations were evident for ERCC1 and EGFR mutation status (Table 8).

**Table 6:** Factors associated with PFS and OS in patients with NSCLC treated with Platinum doublets.

Factor			Median PFS(days)	*P* value^a^	Median OS(days)	*P* value^a^
Age (years)						
High (>63)		25	254.0	0.512	1381.0	0.178
Low (<63)		26	219.0		684.0	
Sex						
Male		19	253.0	0.129	563.0	0.193
Female		32	219.0		1296.0	
Histology						
Adenocarcinoma		45	243.0	0.317	969.0	0.014
Non-adeno		6	297.0		398.5	
Smoking status						
Never-smoker		31	260.0	0.027	1296.0	0.04
Smoker		20	229.0		563.0	
EGFR mutation						
Exon19 deletion		15	303.0	0.057	1450.0	0.146
L858R		12	226.5		596.5	
Negative		24	216.0		714.0	
ERCC1						
Negative		23	281.0	0.039	1083.5	0.487
Positive		28	213.5		684.0	

a Univariate analysis by log-rank test.

**Table 7:** Multivariate analysis of PFS.

Factor	Comparison	HR (95%CI)	P-value
Smoking status	Never smoker/Smoker	0.436 (0.225–0.840)	0.013
ERCC1	Negative/Positive	0.579 (0.312–1.060)	0.076
Mutation	Exon19 deletion/Wild type	0.484 (0.227–0.984)	0.045
	L858R/Wild type	1.050 (0.486–2.163)	0.897

**Table 8:** Multivariate analysis of OS.

Factor	Comparison	HR (95%CI)	P-value
Smoking status	Nevre smoked/smoker	0.437 (0.219–0.880)	0.021
Histology	Adenocarcinoma/Non-adeno	0.346 (0.138–0.985)	0.047
ERCC1	Negative/Positive	0.820 (0.391–1.699)	0.594
Mutation	Exon19 deletion/Wild type	0.685 (0.273–1.664)	0.406
	L858R/Wild type	1.455 (0.625–3.247)	0.372

## Discussion

Prospective clinical trials of EGFR-TKI treatment for NSCLC patients with activating EGFR mutations, such as delE746-A750 (exon 19) and L858R (exon 21), have demonstrated high clinical response rates of approximately 80%. Randomized clinical trials to compare the efficacy of EGFR-TKIs or platinum-based compounds as first-line chemotherapy for patients with NSCLC harboring EGFR mutations [4]–[7] have demonstrated that NSCLC patients with EGFR mutations show a dramatic response to EGFR-TKIs and a longer PFS than those without *EGFR* mutations. Coincident with these results, our present study showed that the median PFS was significantly longer in EGFR mutation-positive than in EGFR mutation-negative patients. In the IPASS study, NSCLC patients with EGFR mutation showed a higher response rate than patients without EGFR mutation when they received carboplatin and paclitaxel. However, PFS did not differ between NSCLC patients with and without EGFR mutations who received cytotoxic agents [8]. These results suggest that NSCLC patients with EGFR mutations may have a tendency to respond to cytotoxic agents such as platinum-based compounds. In accordance with these results, we also found that NSCLC patients harboring EGFR mutations had a higher response rate than patients without EGFR mutations (36.9% vs 17.5%). Lin and Kalikaki et al. have reported that NSCLC patients harboring EGFR mutations show a better response to chemotherapy, whereas other researchers have reported that the presence of EGFR mutations is not correlated with chemotherapy responsiveness [9]–[13]. We then examined the association between EGFR mutation and survival, and found that the median PFS and OS were significantly longer in patients with exon 19 deletion who received platinum-based chemotherapy. Furthermore, Cox regression analysis revealed that EGFR mutation was significantly associated with PFS and OS. There has been some disagreement among researchers with regard to the association between EGFR mutation status and survival after conventional chemotherapy. Hotta et al. demonstrated that EGFR mutation affected PFS, whereas other researchers found no such correlation [9]–[13]. Two previous studies have indicated that NSCLC patients harboring exon 19 deletions seem to be the most responsive to EGFR-TKIs [19]–[20]. Our findings favor the interpretation that chemotherapy is more effective for patients who harbor EGFR mutation than for those who are negative. Further investigations are needed to confirm whether or not EGFR mutation may be predictive of the response to cytotoxic agents.

In order to improve the prognosis of patients with NSCLC, a biomarker that can predict the efficacy of chemotherapy is needed. ERCC1 is a component of the nucleotide excision repair pathway, which is essential for the repair of platinum-DNA adducts. Previous studies have demonstrated that overexpression of ERCC1 tends to be correlated with resistance to platinum-based chemotherapy [14]–[15]. NSCLC patients with low expression of ERCC1 show longer survival after platinum-based chemotherapy than those showing a high ERCC1 expression. Gandara et al. investigated the association between ERFR mutation and ERCC1 mRNA levels in 1207 NSCLC tumor samples, and reported that tumors harboring activating EGFR mutations were more likely to express low levels of ERCC1 mRNA [17]. Re et al. also reported that specimens harboring EGFR mutations were likely to have low expression of ERCC1 [21], and Lee et al., using immunohistochemistry, found a significant correlation between EGFR mutation and low levels of ERCC1 in tumor cells [22]. In agreement with these results, we found a significant correlation between ERCC1 expression and EGFR mutation (P = 0.030). Although the correlation between EGFR mutation and ERCC1 has been unclear, it can be postulated that an impaired capacity for DNA repair and synthesis may be correlated with increased genome instability and tumor mutations [23]–[24]. Oxidative DNA damage and repair contribute to the development of various human pathologies, including malignancies [25]. In previous studies, we have demonstrated that activating mutations of the EGFR in NSCLC are closely associated with a decreased ability to repair DNA damage induced by 8-hydroxy-2′-deoxyguanosine (8-OHdG). One possible mechanism whereby 8-OHdG affects EGFR mutations in NSCLC could be failure of the base excision repair process for elimination of oxidized DNA, thus resulting in augmentation of EGFR mutations and promotion of lung carcinogenesis [25]. It remains to be further studied how ERCC1 could affect mutations of the EGFR gene in lung cancer.

We investigated the association between PFS or OS and EGFR mutation and ERCC1, and found that the median PFS was significantly longer in patients who were ERCC1-negative (P = 0.039). As previous studies have demonstrated, NSCLC patients who are negative for ERCC1 show longer PFS [16]. Although our Cox regression analysis revealed no significant effect of ERCC1 status on PFS or OS, this was a retrospective study and the number of patients was too small to prove any significant associations. Subset analysis demonstrated that ERCC1-negative patients with exon 19 deletion had longer PFS than the others, whereas ERCC1-positive patients without exon 19 deletion had shorter PFS than the others. Interestingly, Gandara et al. found that cancers with exon 19 deletions had a tendency to express lower levels of ERCC1 than cancers with the wild type or L858R [17]. These findings suggest that median PFS and OS are significantly better in patients harboring exon 19 deletion who receive platinum-based chemotherapy. Two previous studies have indicated that NSCLC patients harboring exon 19 deletions seem to be the most responsive to EGFR-TKIs [19]–[20]. Furthermore, prospective clinical trials involving NSCLC patients, such as OPTIMAL and EURTAC, have demonstrated that exon19 patients have a more favorable hazard ratio than those with exon21 mutations. On the other hand, prospective clinical trials of EGFR-TKI treatment for NSCLC patients with *EGFR* mutations, such as NEJ002 and WJTOG3405, have not demonstrated differences in response between patients harboring exon19 and exon21 mutations. Considering this situation, it is still unresolved whether EGFR exon19 deletions are represent a generally positive prognostic factor, irrespective the chemotherapy employed. We favor the interpretation that platinum-based chemotherapy is more effective against ERCC1-negative and exon 19-positive NSCLC.

Whereas most NSCLC patients with *EGFR* mutations benefit from treatment with EGFR-TKIs, their clinical efficacy of EGFR-TKIs differs among such patients, and almost all individuals eventually develop resistance to these drugs. Acquired resistance to EGFR-targeted drugs is one of the major obstacles to further improvement of clinical outcomes in this field. Immunostaining for ERCC1 may be helpful for stratifying NSCLC patients for personalized chemotherapies. Because immunohistochemical detection of ERCC1 protein is simple and feasible in a clinical setting, our findings could be widely applicable in this context. Our study suggests potentially promising treatment options for NSCLC patients who relapse after EGFR-TKI treatment.

There were limitations to the present study. First, the number of patients included was relatively small. Second, the retrospective nature of the study did not allow for a standardized measure of PFS. Third, we adopted the peptide nucleic acid (PNA)-locked nucleic acid (LNA) PCR clamp method to identify the EGFR mutations. This method is able to detect 11 different mutations in the presence of a 100- to 1,000-fold wild-type EGFR background [16]. These 11 mutations account for 95% of all EGFR mutations found in Japanese patients, suggesting that the remaining 5% cannot be detected by the PNA-LNA PCR clamp method [21]. Therefore it is possible some patients in whom mutations were not detected may actually have harbored mutations.

In conclusion, we have found that exon 19 deletion is associated with a longer PFS in NSCLC patients receiving platinum-based chemotherapy. Our findings suggest that platinum-based chemotherapy is more effective against ERCC1-negative and exon 19-positive NSCLC. Further study is warranted to clarify the clinical utility of immunohistochemical analysis of ERCC1 for determination of the optimal anticancer therapeutic regimen using EGFR-targeting drugs.
